# Insulin Receptor Substrate 1 Signaling Inhibits Foxp3 Expression and Suppressive Functions in Treg Cells through the mTORC1 Pathway

**DOI:** 10.3390/ijms24032551

**Published:** 2023-01-29

**Authors:** Woo Ho Lee, Ga Eul Kim, Kyung Jin Hong, Hyeong Su Kim, Gap Ryol Lee

**Affiliations:** Department of Life Science, Sogang University, 35 Baekbeom-ro, Mapo-gu, Seoul 04107, Republic of Korea

**Keywords:** IRS1, Treg cell, Foxp3, mTORC1, colitis

## Abstract

Regulatory T (Treg) cells play an important role in immune homeostasis by inhibiting cells within the innate and adaptive immune systems; therefore, the stability and immunosuppressive function of Treg cells need to be maintained. In this study, we found that the expression of insulin receptor substrate 1 (IRS1) by Treg cells was lower than that by conventional CD4 T cells. IRS1-overexpressing Treg cells showed the downregulated expression of FOXP3, as well as Treg signature markers CD25 and CTLA4. IRS1-overexpressing Treg cells also showed diminished immunosuppressive functions in an in vitro suppression assay. Moreover, IRS1-overexpressing Treg cells were unable to suppress the pathogenic effects of conventional T cells in a transfer-induced colitis model. IRS1 activated the mTORC1 signaling pathway, a negative regulator of Treg cells. Moreover, IRS1 destabilized Treg cells by upregulating the expression of IFN-γ and Glut1. Thus, IRS1 acts as a negative regulator of Treg cells by downregulating the expression of FOXP3 and disrupting stability.

## 1. Introduction

Regulatory T (Treg) cells, a lineage of CD4+ T cells, maintain immune homeostasis [1]. Th1, Th2, and Th17 cells, which are all subsets of conventional CD4 T (Tconv) cells, activate immune responses against pathogens. However, the actions of Treg cells counteract those of Tconv cells, thereby suppressing immune responses and maintaining immune homeostasis [2]. Therefore, the disruption of Treg cell function can trigger autoimmune diseases [3].

CD4+ T cells express key transcription factors that drive differentiation programs and maintain their functions. Th1 cells, which express the transcription factor T-bet, secrete IFN-γ. Th2 cells, which express GATA3, secrete IL-4, IL-5, and IL-13. Th17 cells, which express RORγt, secrete IL-17A and IL17F. Treg cells express the key transcription factor Forkhead box P3 (FOXP3), which induces the expression of CD25, CTLA4, ICOS, and GITR, all of which maintain the immunosuppressive functions and stability of Treg cells [4,5,6]. Foxp3 is used as a lineage-specific marker for identifying and defining Treg cells [4,5,6]. The loss of Foxp3 expression is observed under certain inflammatory conditions, suggesting their lineage stability may not be firmly maintained [4,5,6]. The major function of Treg cells is to inhibit immune cells that are involved in innate and adaptive immune responses [4,5,6,7].

The expression of FOXP3 is regulated by various signaling pathways, including those triggered by T cell receptors (TCRs) and cytokines. Treg cells are generated both in the thymus and in the periphery, and thus are referred to as thymus-derived Tregs (tTregs) or perhphery-derived Tregs (pTregs), respectively. In the thymus, TCR signaling induces the expression of NF-κB and NFAT, and IL-2 signaling induces the expression of STAT5, which binds to CNS2 of the *Foxp3* gene [8,9,10,11,12]. In pTregs, TCR and IL-2 signaling induces the expression of NFAT and STAT5. However, unlike tTregs, these factors bind to CNS1 of the *Foxp3* gene [8]. In addition, TGF-β induces the expression of SMAD3, which also binds to CNS1 of the *Foxp3* gene in pTreg cells [13,14]. 

Although TCR and IL-2 signaling induce the generation of tTreg and pTreg cells, they also activate mammalian target of rapamycin (mTOR) complexes (mTORCs), which are negative regulators of Treg cells [15]. mTOR is induced by immune signals via TCR, cytokines, growth factors, hormones, and nutrients (essential amino acids and glucose) [15,16]. As such, mTOR supports cell survival, growth, and metabolism. It is also important for the differentiation and function of both Tconv cells and Treg cells [17]. There are two types of mTORC: mTORC1 and mTORC2. Typically, mTORC1 is activated by PI3K/AKT and then activates eukaryotic translation initiation factor 4E-binding protein 1 (4EBP1) and ribosomal protein S6 kinase 1 (S6K1) [18]. Thus, mTORC1 acts as a positive regulator of Tconv cells. In contrast, it is a negative regulator of Tregs. Interestingly, the expression of mTORC1 should be downregulated in Treg cells, although it is not blocked completely, because mTORC1 signaling is important for Treg metabolism and stability [19,20,21]. Thus, Treg cells must regulate the mTORC1 pathway precisely.

Insulin receptor (InsR) and insulin-like growth factor 1 (IGF1) receptor (IGF1R) regulate the differentiation and function of Th17 and Treg cells. Insulin inhibits IL-10 production by Treg cells through Akt-mTOR signaling [22]. InsR signaling prevents the accumulation of adipose Treg cells in a high-fat-diet-induced metabolic syndrome model [23]. InsR facilitates the transition of CD73^hi^ST^low^ (immature) into CD73^low^ST2^hi^ (mature) adipose Treg cells [24]. Signaling through IGF1R preferentially promotes differentiation into Th17 cells rather than Treg cells (which differentiate through the Akt/mTOR signaling) [25]. However, the expression of IGF1R is comparable between Th17 and Treg cells, suggesting that mechanisms other than IGF1R signaling are involved in regulating Th17/Treg differentiation. These facts prompted us to investigate the role of another component of insulin/IGF1 signal transduction, insulin receptor substrate 1 (IRS1).

IRS1 is a signaling molecule that acts downstream of InsR or IGF1R [26]. It comprises a highly conserved N-terminal region and a C-terminal region which contains multiple sites that are phosphorylated by PI3K, GRB2, and SHP2 [27]. When IGF1 or insulin bind to IGF1R or InsR, IRS1 is phosphorylated and activates the PI3K/AKT/mTORC1, MAPK, and JAK2/STAT pathways [28,29,30], which in turn trigger cell proliferation, protein synthesis, and transcription.

In this study, we found that IRS1 signaling negatively affects Treg cell stability and function. The expression of IRS1 by Treg cells was much lower than that by Tconv cells. When Treg cells were treated with insulin or IGF1, FOXP3 expression decreased. Treg cells overexpressing IRS1 showed reduced expression levels of FOXP3, CD25, and CTLA4 and had diminished suppressive functions both in vitro and in vivo. IRS1 signaling activated by IGF1 or insulin triggered the mTORC1 signaling pathway, but not the MAPK or JAK2/STAT pathways, in Treg cells. In summary, these results suggest that IRS1 signaling plays a negative role in Treg cells through mTORC1 signaling.

## 2. Results

### 2.1. Treg Cells Show Low Expression of Irs1, and IRS1 Signaling Inhibits Treg Differentiation 

**Figure 1:**
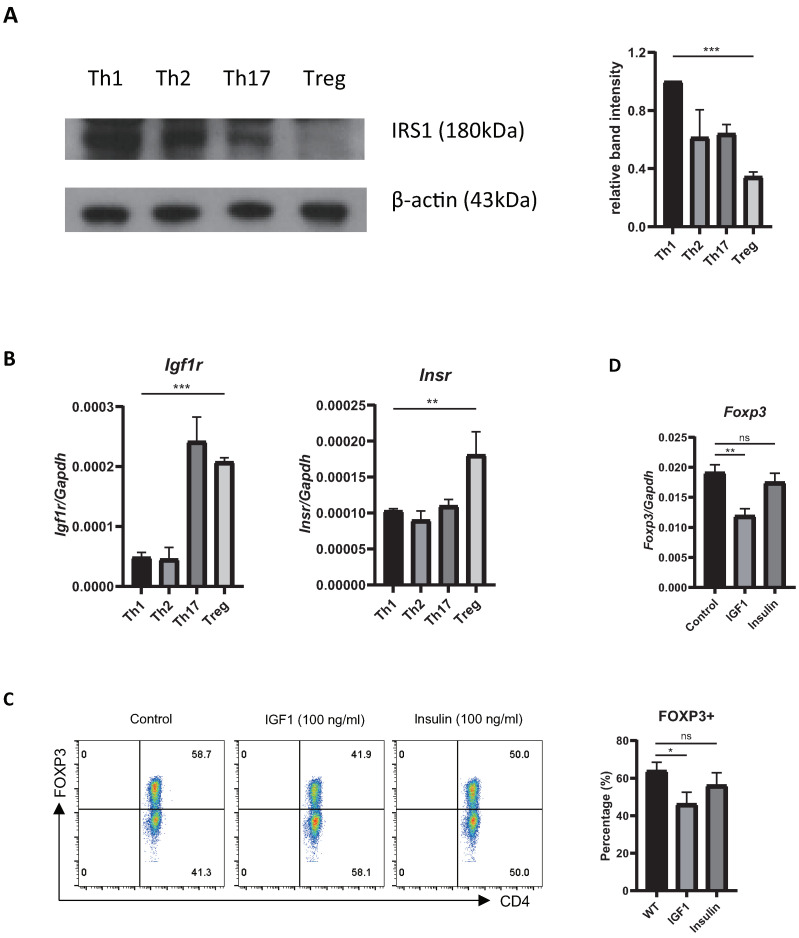
Expression of IRS and IRS-mediated signaling in Treg cells. (**A**) IRS1 was measured in Th1, 2, 17, and Treg cells via immunoblotting. Data were normalized to expression of β-actin. Data were pooled from three independent experiments. (**B**) Naïve CD4 T cells were cultured under Treg-inducing conditions for 3 days, and expression of *Igf1r* and *Insr* mRNA was measured via qRT-PCR. (**C**) Naïve CD4 T cells were cultured under Treg-inducing conditions for 3 days with PBS (control), IGF1, or insulin. FOXP3 protein levels were measured via flow cytometry, and (**D**) *Foxp3* mRNA level was measured via qRT-PCR. Data are representative of three independent experiments. The bar graph next to the flow cytometry data shows data pooled from three independent experiments. The error bars represent the SD, (**A**–**D**) *p*-values were calculated using one-way ANOVA/Tukey test. * *p* < 0.05, ** *p* < 0.01, *** *p* < 0.001. ns: not significant.

To explore the role of IRS1 during Th17/Treg differentiation, we first measured its expression by Tconv cells and Treg cells. We found that IRS1 expression by Treg cells was much lower than that by Tconv cells (Figure 1A). To examine the effects of insulin and IGF1, which are upstream ligands of IRS1 signaling, on Treg cell differentiation, we first examined the expression of the receptors of these ligands in CD4 T cell subsets. The expression of *Igf1r* was much higher in Th17 and Treg cells than in Th1 and Th2 cells, whereas that of *Insr* was slightly higher in Treg cells (Figure 1B). Next, we stimulated Treg cells in vitro with IGF1 and insulin. The FOXP3 expression by these cells was reduced by both IGF1 and insulin; however, the effect of IGF1 was more pronounced (Figure 1C). The expression of *Foxp3* mRNA was also reduced (Figure 1D). These results suggest that IRS1 signaling negatively regulates Treg cell differentiation.

### 2.2. Overexpression or Knockdown of IRS1 Affects Treg Cell Differentiation

**Figure 2:**
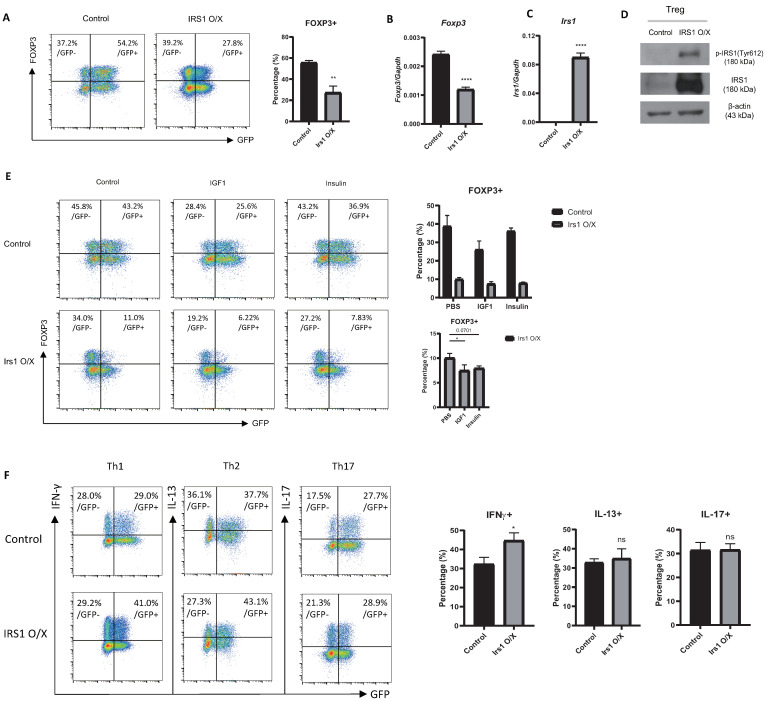
Overexpression of IRS1 reduces Treg cell differentiation. (**A**–**E**) Naïve CD4 T cells isolated from C57BL/6J mice were cultured for 1 day under Th0 conditions and then transduced with the MIEG3-IRS1 vector. After transduction, cells were cultured for 2 days under Treg-inducing conditions. (**A**) FOXP3 protein levels were measured via flow cytometry. (**B**–**D**) GFP+ cells, sorted using a flow cytometer, were used. (**B**) *Irs1* and *Foxp3* mRNA levels were measured via qRT-PCR. (**D**) Y612-phosphorylated form and whole protein of IRS1 were measured via Western blot analysis. β-actin was used as an internal control. (**E**) After transduction, PBS or IGF1 (100 ng/mL) or insulin (100 ng/mL) were treated and cultured for 2 days under Treg conditions. FOXP3 expression was measured via flow cytometry. (**F**) Naïve CD4 T cells were transduced with the MIEG3-IRS1 vector (as in (**A**)). The cells were then differentiated into each subset for 2 days. IFN-γ, IL-13, and IL-17 proteins were measured via flow cytometry. Expression was measured via qRT-PCR and normalized to that of *Gapdh*. All data are representative of three independent experiments. The bar graph next to the flow cytometry plot shows data pooled from three independent experiments. The error bars represent the SD, and (**A**–**C**,**F**) *p*-values were calculated using a *t*-test. (**E**) *p*-value of the bottom graph was calculated using one-way ANOVA/Tukey test. * *p* < 0.05, ** *p* < 0.01, **** *p* < 0.0001. ns: not significant.

To further investigate the role of IRS1 on Treg cell differentiation, we performed an RV-mediated overexpression experiment. When Treg cells were transduced with an IRS1-overexpressing construct (MIEG3-IRS1), a portion of Treg cells lost the expression of Foxp3 (Figure 2A); the expression of *Foxp3* mRNA was also reduced (Figure 2B). The overexpression of IRS1 by these cells was confirmed at both the mRNA and protein levels (Figure 2C,D). The Y612-phosphorylated form of IRS1, an active form of IRS1 [31], was increased in IRS1-overexpressing cells (Figure 2D). The treatment of IGF1 or insulin or the overexpression of IRS1 reduced the Foxp3 expression in Treg cells (Figure 2E). When IRS1 overexpression and the treatment of IGF1/insulin were combined, it further reduced the Foxp3 expression (Figure 2E), indicating that overexpressed IRS1 can transmit IGF1/insulin signals. We also examined the effect of IRS1 overexpression on effector subsets of CD4 T cells (Th1, Th2, and Th17 cells). IRS1 overexpression induced Th1 cell differentiation, but not Th2 and Th17 differentiation (Figure 2F), suggesting that the negative regulatory effects of IRS1 are specific to Treg cells.

**Figure 3:**
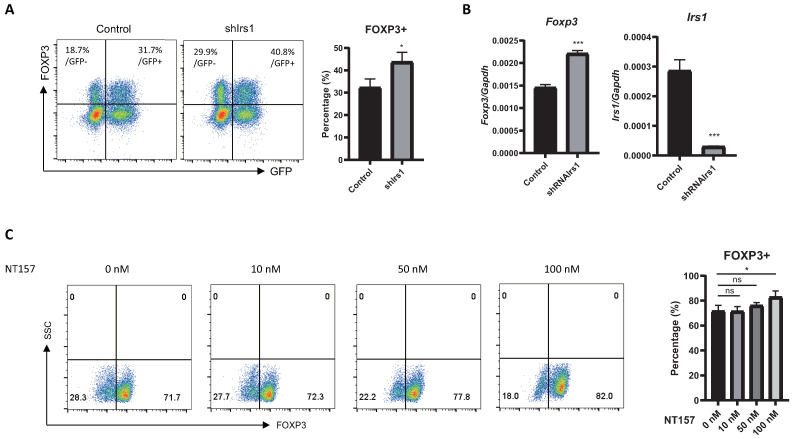
Knockdown or pharmacological inhibition of IRS1 enhances Treg cell differentiation. (**A**) Naïve CD4 T cells isolated from C57BL/6J mice were cultured for 1 day under Th0 conditions and then transduced with the shRNA-Irs1 vector. Cells were then cultured for 2 days under suboptimal Treg-inducing conditions. GFP+ cells were used for transduction. FOXP3 expression was measured via flow cytometry. (**B**) *Irs1* and *Foxp3* mRNA levels. Expression was measured via qRT-PCR and normalized to that of *Gapdh*. (**C**). Naïve CD4 T cells were cultured under Treg-inducing conditions in the presence of NT157 for 3 days, and the percentages of Foxp3-expressing cells were measured via flow cytometry. All data are representative of three independent experiments. The bar graph next to the flow cytometry plot shows data pooled from three independent experiments. The error bars represent the SD, and (**A**,**B**) *p*-values were calculated using a *t*-test. (**C**) *p*-values were calculated using one-way ANOVA/Tukey test. * *p* < 0.05, *** *p* < 0.001. ns: not significant.

Next, we examined the effect of the shRNA-mediated knockdown of IRS1 on Treg cell differentiation. For this, we cultured naïve CD4 T cells under suboptimal Treg-inducing conditions to avoid the saturation of FOXP3 expression. IRS1 knockdown promoted the expression of Foxp3 by Treg cells (Figure 3A). We confirmed a great reduction in the expression level of *Irs1* mRNA, as well as the increased expression of *Foxp3* mRNA, via RT-PCR (Figure 3B). We further examined the effect of IRS1 inhibition by treating Treg cells with a specific pharmacological inhibitor, NT157. The Foxp3 expression was dose-dependently increased with NT157 treatment (Figure 3C), consistent with the knockdown experiment. Collectively, these data suggest that IRS1 functions as a negative regulator of Treg cells.

### 2.3. IRS1 Downregulates Expression of Treg Signature Genes, as Well as Suppressive Activity

**Figure 4:**
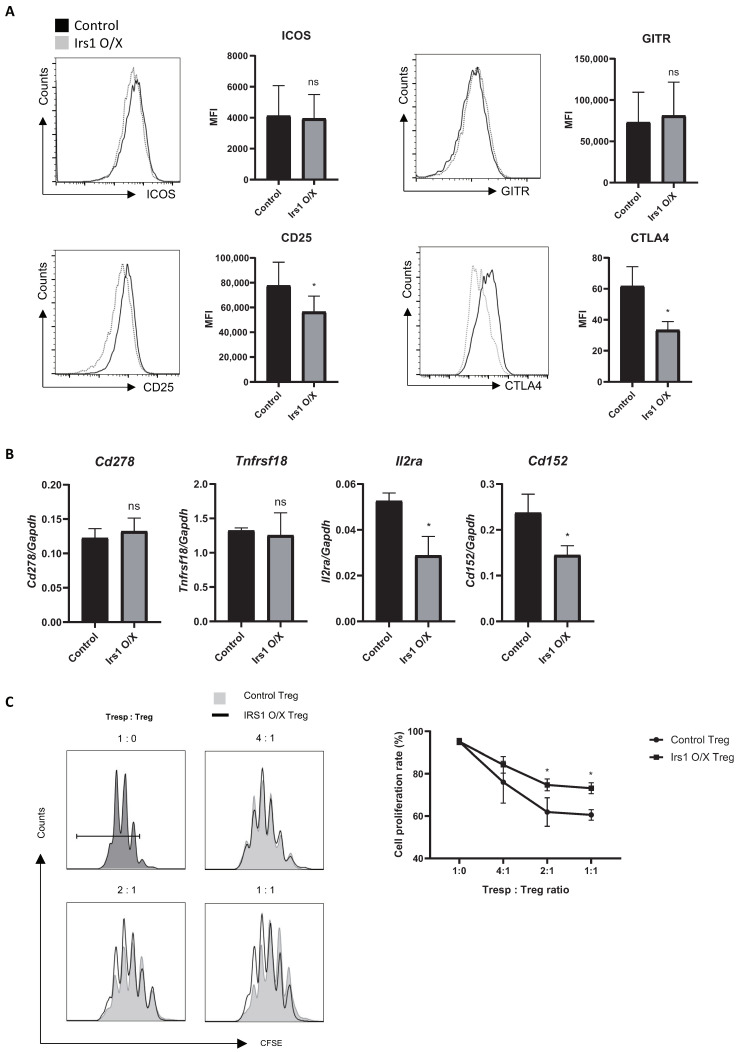
IRS1 downregulates CD25 and CTLA4 on Treg cells and suppresses their immunosuppressive functions. IRS1 overexpression was performed as in Figure 2. (**A**) GFP+ Treg cells (transduced by MIEG3-IRS1) were gated, and expression of ICOS, GITR, CD25, and CTLA was measured via flow cytometry. (**B**) RNA was isolated from GFP+ cells. Expression of mRNA encoding ICOS, GITR, CD25, and CTLA4 was measured via qRT-PCR. (**C**) Thy1.1 + CFSE-labeled responder CD4 T cells were mixed with IRS1-overexpressing Treg cells in various ratios and then cultured for 3 days with anti-CD3/CD28 beads. After 3 days, responder CD4 T cells were stained with a Thy1.1 antibody and then analyzed via flow cytometry. All data are representative of three independent experiments. The bar graph next to the flow cytometry plot shows data pooled from three independent experiments. The error bars represent the SD, and (**A**,**B**) *p*-values were calculated using a *t*-test. (**C**) *p*-values were calculated using two-way ANOVA/Tukey test * *p* < 0.05. ns: not significant.

To further investigate the effects of IRS1 on Treg cells, we measured the expression of Treg cell markers. ICOS (*Cd278*) is required for the suppressive activity of Treg cells, which maintain immune homeostasis [4,32]. GITR (*Tnfrsf18*) is expressed by activated Treg cells, and its expression correlates with their immunosuppressive functions [33]. CD25 (IL-2 receptor α-chain) is important for Treg cell survival and suppressive activity [34], whereas CTLA4 (*Cd152*) intercepts costimulatory signals (B7.1 and B7.2) from antigen-presenting cells and blocks effector T cell activation [35,36]. Thus, these markers are important for the immunosuppressive activity of Treg cells. IRS1 overexpression by Treg cells downregulated CD25 and CTLA4, but not ICOS and GITR (Figure 4A). The expression of mRNA encoding ICOS, GITR, CD25, and CTLA4, as measured via qRT-PCR (Figure 4B), was consistent with the expression levels of the respective proteins. To further examine the effect of IRS1 on Treg cell function, we performed an in vitro suppression assay. Naïve CD4 T cells were mixed with control or IRS1-overexpressing Treg cells, and their proliferation was measured. The results showed that the proliferation of naïve CD4 T cells was less inhibited by IRS1-overexpressing Treg cells than by control Treg cells (Figure 4C), suggesting that IRS1 signaling inhibits the immunosuppressive function of Treg cells.

### 2.4. IRS1-Overexpressing Treg Cells Fail to Control Colitis

**Figure 5:**
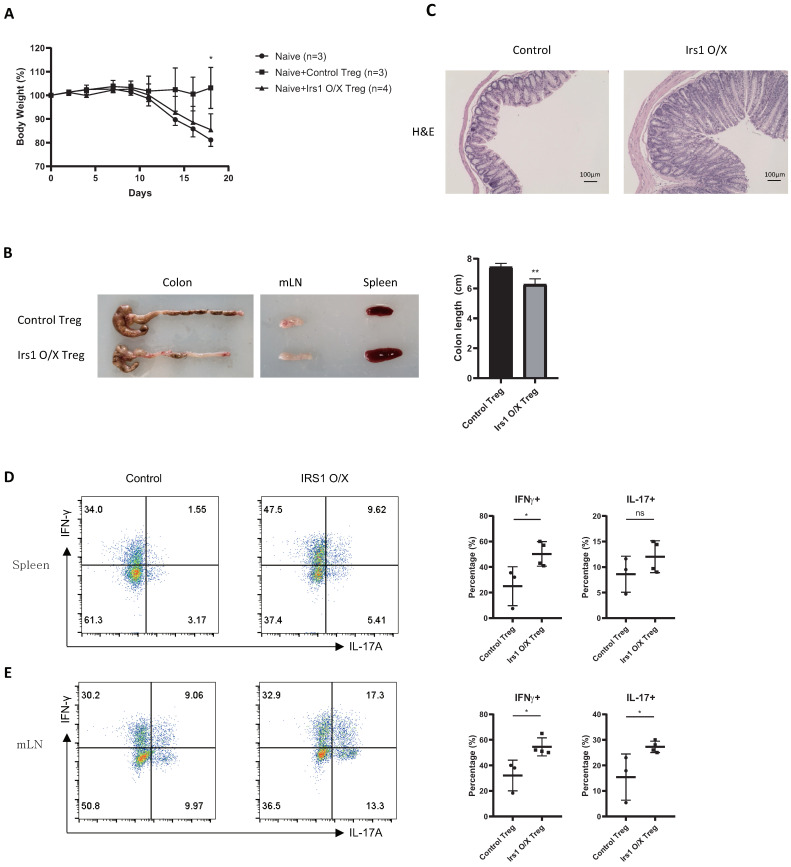
The suppressive function of IRS1-overexpressing Treg cells is impaired in vivo. (**A**) Body weight of *Rag1* KO mice was measured after injection of naïve CD4 T cells (5 × 10^5^), either alone or together with Treg cells (1 × 10^5^) (n = 3 or 4 for each group). (**B**) Morphology of the colon, mLN, and spleen. (**C**) Histology of the clone stained using H & E. (**D**,**E**) IFN-γ^+^ or IL-17^+^ cells (CD4^+^ gated population) in the spleen (**D**) or mesenteric lymph nodes (**E**) were measured via flow cytometry. The bar graph next to the flow cytometry plot shows data pooled from three independent experiments. The error bars represent SD, and *p*-values were analyzed via *t*-test. * *p* < 0.05, ** *p* < 0.01. ns: not significant.

To examine the role of IRS1 in Treg cells in vivo, we performed transfer-induced colitis experiments. FACS-sorted naïve CD4 T cells were adoptively transferred into *Rag1*-deficient mice in the presence or absence of control Treg cells or IRS1-overexpressing Treg cells. Control mice that received naïve CD4 T cells only lost weight continuously. In contrast, mice that received naïve CD4 T cells + control Treg cells did not; however, this effect was abolished in mice that received naïve CD4 T cells + IRS1-overexpressing Treg cells (Figure 5A). Mice receiving naïve CD4 T cells + IRS1-overexpressing Treg cells also had shorter colons, developed splenomegaly, and had thicker epithelial layers (Figure 5B,C). Moreover, the percentage of IFN-γ- and/or IL-17A-secreting cells in the spleen and mesenteric lymph nodes (mLNs) was increased (Figure 5D,E). These results suggest that IRS1 inhibits Treg function under physiological conditions.

### 2.5. IRS1 Induces mTORC1 Pathway, but Not the MAPK and JAK2/STAT Pathways, in Treg Cells

**Figure 6:**
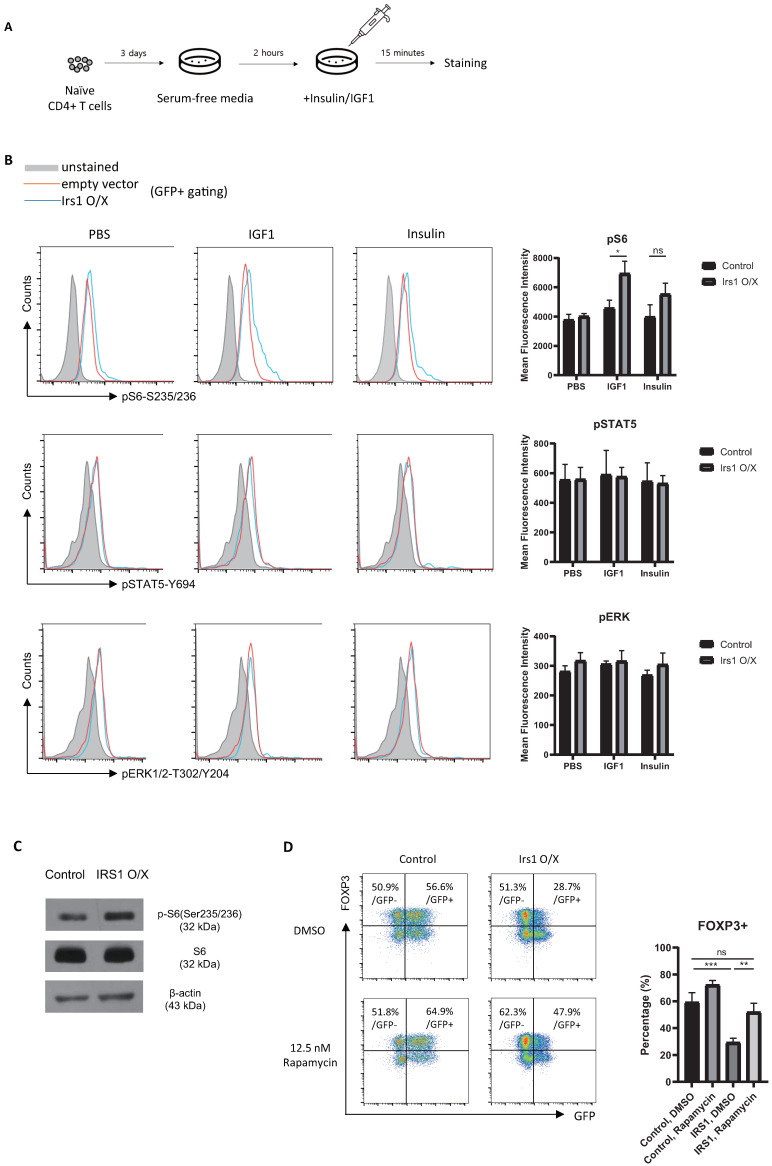
IRS1 signaling activates the mTORC1, but not the JAK/STAT and ERK pathways. (**A**) Schematic of the signaling activation experiments. (**B**,**C**) Signaling molecules were stained with appropriate antibodies, and cells were analyzed via flow cytometry (**B**) and via Western blot analysis (**C**). (**D**) Naïve CD4 T cells were cultured for 1 day under Th0 conditions and then transduced with the MIEG3-IRS1 vector. Transduced cells were cultured for 2 days under Treg-inducing conditions in the presence of DMSO (control) or rapamycin. The cells were then analyzed via flow cytometry. All data are representative of three independent experiments. The bar graph next to the flow cytometry plot shows data pooled from three independent experiments. The error bars represent the SD, and (**B**) *p*-values were calculated using a *t*-test. (**D**) *p*-values were calculated using one-way ANOVA/Tukey test. * *p* < 0.05, ** *p* < 0.01, *** *p* < 0.001. ns: not significant.

To explore the molecular mechanisms by which IRS1 regulates Treg cell differentiation and function, we investigated signaling molecules associated with IRS1. IRS1 is activated not only by InsR and IGF1R, but also by cytokine receptors and hormone receptors [29]. When IRS1 is activated by various receptors, it transmits signals through the AKT/mTORC1, MAPK, and JAK2/STAT pathways in a cell-context-dependent manner [28,29,30]. Therefore, we examined which signaling pathway in Treg cells is used for IRS1 signaling by insulin or IGF1. Because these ligands are present in FBS, we sought to minimize their effects on Treg cell signal transduction by culturing Tregs in a serum-free medium for 2 h. After exposure to insulin or IGF1 for 15 min, cells were harvested, and the phosphorylation status of various signaling molecules was examined (Figure 6A). Interestingly, the phosphorylation of S6 kinase in Treg cells increased upon exposure to both insulin and IGF1 (Figure 6B,C). The JAK/STAT5 pathway induces the expression of FOXP3 by Treg cells [37], and STAT5 is activated by IGF1/IGF1R in growth hormone-stimulated liver cells [38,39]. However, we observed no change in STAT5 expression by Treg cells in response to IGF1 or insulin, even when IRS1 was overexpressed (Figure 6B); this suggests that IRS1 signaling is context-dependent. ERK signaling downregulated the expression of FOXP3 by Treg cells in a mouse model of colitis [40]. However, as with STAT5, ERK signaling in iTreg cells was not activated by IGF1/IRS1 signaling (Figure 6B). To confirm that FOXP3 is regulated by the mTORC1 pathway (which is activated by IRS1), we treated IRS1-overexpressing Treg cells with an mTORC1 inhibitor, rapamycin. When IRS1-overexpressing Treg cells were cultured with rapamycin, FOXP3 expression was restored (Figure 6D). The expression of FOXP3 by control Treg cells did not increase as much as in IRS1-overexpressing Treg cells treated with rapamycin. These data suggest that IRS1 signals induced by insulin and IGF1 to downregulate FOXP3 expression by Treg cells are transduced through mTORC1, but not through JAK/STAT and ERK.

### 2.6. The IGF1/IRS1 Pathway Affects Treg Cell Stability and Upregulates IFN-γ and Glucose Uptake

**Figure 7:**
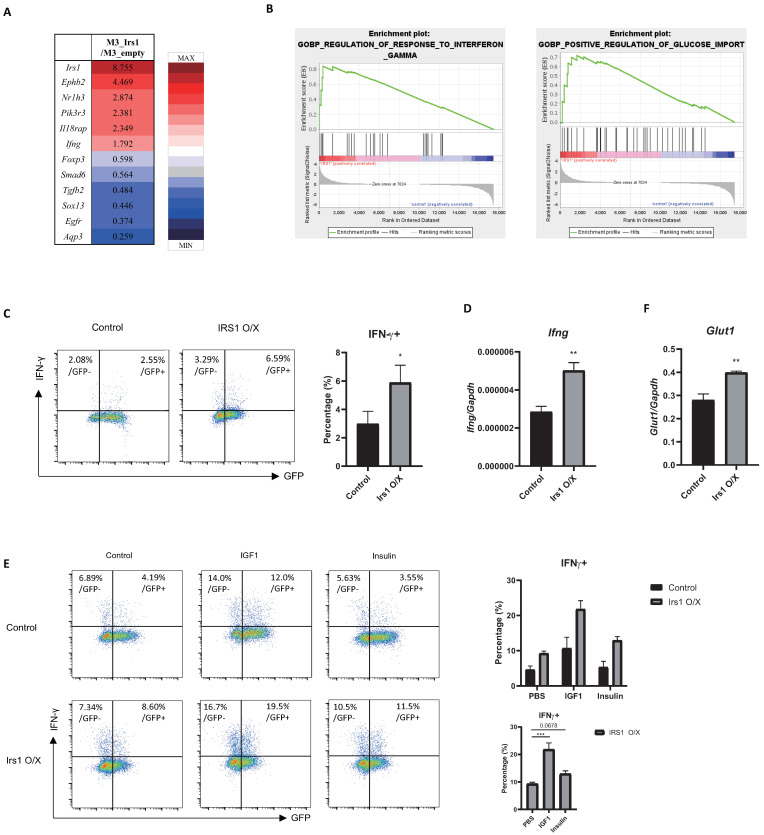
IRS1 signaling affects the stability and metabolism of Treg cells. (**A**) Heatmap of genes showing altered expression in IRS1-overexpressing Treg cells compared with controls. (**B**) GSEA analysis of “*Ifng*-associated genes” and “glucose uptake-associated genes” in control and IRS1-overexpressing Treg cells. (**C**) IFN-γ expression in IRS1-overexpressing Treg cells was measured via flow cytometry. Cells were gated on FOXP3+, and the ratio of IFN-γ^+^ cells to GFP- or GFP+ cells was measured. (**D**) RNA was isolated from sorted GFP+ cells, and expression of *Ifng* was measured via qRT-PCR. (**E**) Retroviral transduction of MIEG3-IRS1 vector was performed as in Figure 2. After transduction, PBS or IGF1 (100 ng/mL) or insulin (100 ng/mL) were treated and cultured for 2 days under Treg conditions. IFN-γ expression was measured via flow cytometry. (**F**) RNA was isolated from sorted GFP+ cells, and *Glut1* was measured via qRT-PCR. Flow cytometry and qRT-PCR data are representatives of three independent experiments. The bar graph next to the flow cytometry plot shows that data were pooled from three independent experiments. The error bars represent the SD, and (**C**,**D**,**F**) *p*-values were calculated using a *t*-test. (**E**) *p*-value of the bottom graph was calculated using one-way ANOVA/Tukey test. * *p* < 0.05, ** *p* < 0.01, *** *p* < 0.001.

To study changes in global gene expression induced by IRS1 signaling, we performed RNA-sequencing (RNA-seq) analysis of control and IRS1-overexpressing Treg cells. We noted changes in the expression of many genes upon the overexpression of IRS1 (Figure 7A); the decline in the expression of *Foxp3* was particularly notable. To identify the pathways or groups of genes that are altered specifically by IRS1, we performed gene set enrichment analysis (GSEA). We found that gene sets involved in the “regulation of response to interferon-gamma” and the “positive regulation of glucose import” were enriched in IRS1-overexpressing Treg cells (Figure 7B). In addition, RNA-seq data showed that *Ifng* was upregulated in IRS1-overexpressing Treg cells (Figure 7A). Interestingly, IRS1 overexpression also increased the expression of IFN-γ by Th1 cells (Figure 2). To confirm the RNA-seq data, we measured the IFN-γ expression in IRS1-overexpressing Treg cells. IRS1 overexpression supported the expression of IFN-γ protein and mRNA in Treg cells (Figure 7C,D). When IRS1-overexpressing cells were treated with IGF1 or insulin, they produced much more IFN-γ than untreated cells did (Figure 7E). These results suggest that the IRS1/mTORC1 pathway in Treg cells downregulates FOXP3 and promotes the expression of proinflammatory cytokines. A previous study showed that IRS1 is involved in glucose uptake stimulated by insulin signaling [41]. Th1, 2, and 17 effector T cells prefer to use glycolysis for metabolism, while Treg cells prefer to use fatty acid oxidation [42]. Additionally, the upregulation of glycolysis disrupts Treg cells and interferes with their immunosuppressive function [43]. To confirm the GSEA data, we measured the expression of *Glut1*, a major glucose transporter, and found that it was upregulated in IRS1-overexpressing Treg cells (Figure 7F). These data suggest that IRS1 signaling alters the metabolic pathway in Treg cells from fatty acid metabolism to glycolysis, which may lead to Treg cell instability.

## 3. Discussion

In this study, we examined the role of IRS1 signaling in Treg cell differentiation and function. IRS1 inhibited the differentiation and suppressive functions of Tregs. IRS signaling induced by the binding of insulin and IGF1 to their receptors stimulated mTORC1 activity and reduced the expression of Foxp3 and related Treg markers. The data suggest a previously unidentified role for IRS1 in Treg cells.

We found that IFN-γ was upregulated by IRS1 signaling not only in Treg cells but also in Th1 cells. The molecular mechanism underlying IRS1-mediated IFN-γ expression by T cells is not yet clear. Previous studies show that *Nrp-1* or *Il12rb2* affect IFN-γ expression by Treg cells [44,45]; however, our RNA-seq data revealed no changes in such factors in IRS1-overexpressing Treg cells. In addition, *Tbx21*, a key regulator of IFN-γ, was unchanged in IRS1-overexpressing Treg cells. We speculate that the mTORC1-mediated expression of IFN-γ by Treg cells may be related to the induction of Th1 cell differentiation. 

We also found that the expression of *Glut1* by Treg cells increased when IRS1 was overexpressed, suggesting that IRS regulates glucose uptake by Treg cells. Since the glucose metabolism plays a critical role in diabetes, and Treg cells regulate inflammatory conditions in adipose tissues, it is reasonable to speculate that IRS1 may play a role in regulating Treg cell function under diabetic conditions. Indeed, IRS1 plays a role in brown adipocyte Treg cells. Treg cells reside in adipose tissues, and inflammation induced within adipose tissues correlates with insulin resistance and type 2 diabetes [46,47]. Thus, the roles of IRS1 in adipose tissue Treg cells, and in the development of diabetes, warrant further study.

mTORC1 plays an important role in deciding T cell fate [17]. Signaling through mTORC1 detects changes in the microenvironment around T cells. mTORC1 plays different roles in effector T cells and Treg cells. For example, the upregulation of mTORC1 is essential for effector T cell development and activation, whereas it is downregulated, although not completely blocked, in Treg cells. When mTORC1 is deleted from Treg cells, this affects their stability and immunosuppressive function, resulting in autoimmune disease [21]. Thus, mTORC1 signaling in Treg cells needs to be tightly regulated. IRS1 is activated by the sensing of growth factors or insulin by several receptors. Here, we showed that IRS1 signaling initiated by insulin or IGF1 activated mTORC1 in Treg cells. This signaling plays a role in the downregulation of FOXP3 and the upregulation of glycolysis, both of which are associated with Treg cell instability. IRS1 signaling activates the AKT/mTORC1, MAPK, and JAK/STAT pathways in various cell types [28,29,30]. However, here, we found that IRS1 only upregulates mTORC1 in Treg cells. Given the tight regulation of mTORC1 in Treg cells, it is expected that various factors may regulate mTORC1 signaling [21]. Our findings suggest that IRS1 is a specific regulator of mTORC1 signaling in Treg cells and that the mTORC1 pathway, but not the MAPK or JAK/STAT pathways, is a major mediator of IRS1 signaling in Treg cell differentiation. IRS1 signaling is expected to activate mTORC1 by sensing various factors in the environment. This regulation may be important for maintaining the identity of Treg cells by preventing the excessive activation of mTORC signaling, which destabilizes them. 

In summary, our study identified a role for IRS1 in Treg cell differentiation and function. These findings may help us to understand how Treg cells integrate environmental signals to regulate their status and maintain tissue homeostasis. 

## 4. Materials and Methods

### 4.1. Mice

Female C57BL/6J mice aged 6–12 weeks were used for all experiments. WT mice were purchased from Daehan Bio Link (Eumsung, Republic of Korea). Thy1.1 mice were purchased from The Jackson Laboratory (Bar Harbor, ME, USA). All mice were housed under specific pathogen-free conditions, and all animal experiments were approved by the Sogang University Institutional Animal Care and Use Committee (approval no. IACUCSGU2019_09).

### 4.2. Isolation of CD4+ T Cells and In Vitro Differentiation 

Naïve CD4+ T cells were isolated from mouse spleens using the MojoSort^TM^Ms CD4+ naïve T cell Isolation Kit (BioLegend, San Diego, CA, USA), as described previously [48]. Briefly, cells were cultured in RPMI-1640 medium supplemented with 10% fetal bovine serum (FBS; Gibco, Waltham, MA, USA), MEM amino acids (Gibco, Waltham, MA, USA), non-MEM amino acids (Gibco, Waltham, MA, USA), and penicillin–streptomycin (Gibco, Waltham, MA, USA). Cells were activated by plate-bound anti-CD3 (10 µg/mL) and soluble anti-CD28 (2 µg/mL) antibodies. The culture conditions were as follows: Th0, IL-2 (1 ng/mL, R&D Systems, Minneapolis, MN, USA) for 3 days; Th1, anti-IL-4 antibody (5  μg/mL), mouse recombinant IL-2 (1 ng/mL), and mouse recombinant IL-12 (3.5 ng/mL) (all eBioscience, San Diego, CA, USA); Th2, anti-IFN-γ antibody (5 μg/mL), mouse recombinant IL-2 (1 ng/mL), and mouse recombinant IL-4 (20 ng/mL) (all from R&D Systems, Minneapolis, MN, USA); Th17, anti-IL-4 antibody (10 μg/mL), anti-IFN-γ antibody (5 μg/mL), mouse recombinant IL-6 (50 ng/mL) (all from eBioscience), human recombinant TGF-β1 (2 ng/mL, R&D Systems, Minneapolis, MN, USA), mouse recombinant IL-1β (2 ng/mL; eBioscience, San Diego, CA, USA), and mouse recombinant TNF-α (1 ng/mL; eBioscience, San Diego, CA, USA); and iTregs, anti-IL-4 antibody (5 μg/mL), anti-IFN-γ antibody (5 μg/mL), mouse recombinant IL-2 (0.4 ng/mL) (R&D), and human recombinant TGF-β1 (5 ng/mL) (R&D Systems, Minneapolis, MN, USA). All subsets were cultured for 3 days. Suboptimal iTReg culture conditions were as follows: anti-IL-4 antibody (5 μg/mL), anti-IFN-γ antibody (5 μg/mL), and human recombinant TGF-β1 (0.5 ng/mL).

For some experiments, human recombinant insulin-like growth factor (IGF)1 (Cell Signaling Technology) and human insulin (Sigma) were solubilized in 1× PBS supplemented with 10% bovine serum and then added to the culture medium for 3 days.

### 4.3. Immunoblot Analysis

RIPA buffer (Sigma, St. Louis, MO, USA) containing Protease Inhibitor Cocktail (GenDEPOT) was used for cell lysis. Cell lysates were boiled with lane markers in reducing sample buffer (Thermo Fisher Scientific, Waltham, MA, USA). The proteins in cell lysates were separated via SDS/polyacrylamide gel electrophoresis and then transferred to a PVDF membrane. The membrane was then blocked for 1 h with 5% skim milk/Tris-buffered saline and Tween 20 (TBST). Next, the membrane was incubated overnight at 4 °C with a primary antibody (diluted 1:1000 in 5% skim milk/TBST). The membrane was washed three times with TBST buffer and then incubated for 1 h at room temperature with an HRP-conjugated secondary antibody (1:5000 in 5% skim milk/TBST). Finally, the membrane was washed three times with TBST buffer and incubated with West-Q Pico ECL solution and West-Q Femto clean ECL solution (GeneDEPOT, Baker, TX, USA). Anti-IRS1 (Cell Signaling Technology, Trask Lane Danvers, MA, USA) and anti-β-actin (Santa Cruz Biotechnology, Dallas, TX, USA) were used as primary antibodies. HRP-conjugated antirabbit IgG or HRP-conjugated anti mouse IgG were used as secondary antibodies.

### 4.4. Intracellular Staining

Naïve CD4+ T cells were cultured for 3 days under the appropriate conditions for each subset. Next, cells were stimulated for 4 h with PMA (50 ng/mL) and ionomycin (1 μM, Sigma, St. Louis, MO, USA) in the presence of Brefeldin A (BioLegend, San Diego, CA, USA). After stimulation, cells were fixed in BD Cytofix/Cytoperm^TM^ buffer or FOXP3 Fix/Perm buffer and then permeabilized with BD Perm/Wash^TM^ or FOXP3 Perm Buffer. The following antibodies were used for staining: PE-conjugated anti-IFN-γ (Biolegend, San Diego, CA, USA), PE-conjugated anti-IL-1 (BioLegend, San Diego, CA, USA), PE-conjugated anti-IL-17 (BioLegend, San Diego, CA, USA), APC-conjugated anti-FOXP3 (eBioscience, San Diego, CA, USA), PE-conjugated anti-CD25 (BioLegend, San Diego, CA, USA), PE-conjugated anti-ICOS (BioLegend, San Diego, CA, USA), PE-conjugated anti-GITR (BioLegend), PE-conjugated anti-CTLA4 (BioLegend, San Diego, CA, USA), PE-conjugated anti-ERK1/2 phospho (T302/Y204) (BioLegend, San Diego, CA, USA), PE-conjugated anti-AKT phospho (T308) (BD Biosciences, Franklin Lakes, NJ, USA), APC-conjugated anti-S6 phospho (S235/236) (Invitrogen, Carlsbad, CA, USA), and PercP-conjugated anti-STAT5 (Y694) (BD Biosciences, Franklin Lakes, NJ, USA). Cells were analyzed using a BD FACSCalibur or a BD Accuri C6 Plus cytometer. FlowJo software was used for data analysis and to draw graphs.

### 4.5. Retroviral (RV) Transduction

The *Irs1* coding sequence was used to generate the overexpression and knockdown vectors MIEG3-IRS1 and MSCV-LMP-*Irs1*, respectively. First, phoenix cells (1.2 × 10^6^ cells) were transfected with MIEG3 or MSCV-LMP (2 μg) along with a PCL-Eco helper vector (1 μg). After 2 days, the supernatants were passed through a 0.45 μM syringe filter and then added to polybrene (final concentration, 4 μg/mL) (Sigma, St. Louis, MO, USA). Naïve CD4+ T cells were cultured for 1 day under Th0 conditions. Then, the Th0 medium was removed and replaced with 1 mL of the prepared supernatants. After that, CD4+ T cells were transduced using the spin-down method (1600× *g*, 90 min, 25 °C). After spin-down, the supernatants were removed and replaced with an appropriate medium for Th cell differentiation; cells were then cultured for 2 days. For some experiments, the culture medium was supplemented with DMSO (negative control) or rapamycin (1:1000).

### 4.6. RNA Isolation and Quantitative Real-Time Polymerase Chain Reaction (qRT-PCR)

RNA from Th cells cultured for 3 days was isolated using Trizol reagent (Molecular Research Center, Cincinnati, OH, USA). Next, cDNA was synthesized using TopScript RT (Enzynomics), and a qRT-PCR was performed using TOPreal™ qPCR 2X PreMIX SYBR green (Enzynomics Daejeon, Republic of Korea), TOPreal™ qPCR 2X PreMIX Taqman probe (Enzynomics Daejeon, Republic of Korea), and a Roche LightCycler 96. The primers used for qRT-PCR are listed in the Supplementary Information Table S1.

### 4.7. In Vitro Suppression Assay

To obtain responder cells, naïve CD4 T cells isolated from Thy1.1 mice were stained with CFSE (Sigma, St. Louis, MO, USA). To obtain Treg cells, naïve CD4 T cells isolated from C57BL/6 mice were differentiated into Treg cells for 3 days, as described in the “4.5 retroviral (RV) transduction” section, and GFP+ cells were sorted using a FACS ARIA III cytometer (BD Bioscience, Franklin Lakes, NJ, USA). CFSE-labeled naïve CD4 T cells (8.0 × 10^4^ cells) were mixed with GFP+ Treg cells in a 96-well plate in the presence of anti-CD3/CD28 beads (Dynabeads™ Mouse T-Activator CD3/CD28, Invitrogen, Carlsbad, CA, USA). After 3 days, the cells were stained with an Alexa Fluor 647-conjugated Thy1.1 antibody (Biolegend, San Diego, CA, USA) to distinguish responder cells from Treg cells. CFSE-stained Thy1.1+ cells were analyzed using BD Accuri C6 Plus cytometry. 

### 4.8. Inflammatory Bowel Disease Model

CD4 + CD62L + CD45RB^high^ naïve CD4 T cells were sorted from the spleen of C57BL/6 mice, mixed at a 5:1 ratio with control or IRS1-overexpressing Treg cells, and injected intraperitoneally into *Rag1* KO mice (n = 3 or 4 for each group). Body weight was measured three times a week, and mice were sacrificed on day 18 post-injection. Cells were sorted from the spleen and mLNs and then stimulated for 4 h with PMA and ionomycin in the presence of Brefeldin A. Cells were stained with CD4, IFN-γ, and IL-17A antibodies and then subjected to flow cytometry analysis. Colons were excised, fixed in formalin, and stained with hematoxylin and eosin.

### 4.9. Phosphorylation Analysis

Naïve CD4 T cells were cultured and transduced with RV, as described above. After 3 days, cells were washed with PBS and then incubated in serum-free medium for 2 h. Next, cells were treated for 15 min with PBS (control), IGF1, or insulin. Finally, cells were collected and analyzed with ICS, as described above.

### 4.10. RNA-Seq and Data Analysis

iTreg cells were collected and lysed in Trizol reagent prior to transport to eBiogen Inc. (Seoul, Republic of Korea) for further analysis. Data were analyzed and organized using the ExDEGA program (eBiogen Inc., Seoul, Republic of Korea). Gene classification was based on the Medline database (http://www.ncbi.nlm.nih.gov/, accessed on 9 March 2021). GSEA from the Broad Institute (http://www.gsea-msigdb.org/gsea/index.jsp, accessed on 19 May 2021) was used to calculate the enrichment of genes.

### 4.11. Statistical Analysis

Data are expressed as the mean ± standard deviation (SE). Differences between groups were determined using Student’s *t*-test. When comparing more than two groups, one-way ANOVA or two-way ANOVA was used, and Tukey was used for post-test. *p* values < 0.05 were considered statistically significant (* *p* < 0.05, ** *p* < 0.01, and *** *p* < 0.001).

## Data Availability

The original contribution presented in the study are included in the article. Further inquiries can be directed to the corresponding author.

## Supplementary Materials

The following supporting information can be downloaded at: https://www.mdpi.com/article/10.3390/ijms24032551/s1.

## References

[1] Zhu J., Yamane H., Paul W.E. (2010). Differentiation of effector CD4 T cell populations (*). Annu. Rev. Immunol..

[2] Sakaguchi S., Yamaguchi T., Nomura T., Ono M. (2008). Regulatory T cells and immune tolerance. Cell.

[3] Zhou X., Bailey-Bucktrout S.L., Jeker L.T., Penaranda C., Martinez-Llordella M., Ashby M., Nakayama M., Rosenthal W., Bluestone J.A. (2009). Instability of the transcription factor Foxp3 leads to the generation of pathogenic memory T cells in vivo. Nat. Immunol..

[4] Herman A.E., Freeman G.J., Mathis D., Benoist C. (2004). CD4+CD25+ T regulatory cells dependent on ICOS promote regulation of effector cells in the prediabetic lesion. J. Exp. Med..

[5] Hori S., Nomura T., Sakaguchi S. (2003). Control of regulatory T cell development by the transcription factor Foxp3. Science.

[6] Zheng Y., Josefowicz S.Z., Kas A., Chu T.T., Gavin M.A., Rudensky A.Y. (2007). Genome-wide analysis of Foxp3 target genes in developing and mature regulatory T cells. Nature.

[7] Lee W., Lee G.R. (2018). Transcriptional regulation and development of regulatory T cells. Exp. Mol. Med..

[8] Sharabi A., Tsokos M.G., Ding Y., Malek T.R., Klatzmann D., Tsokos G.C. (2018). Regulatory T cells in the treatment of disease. Nat. Rev. Drug Discov..

[9] Zheng Y., Josefowicz S., Chaudhry A., Peng X.P., Forbush K., Rudensky A.Y. (2010). Role of conserved non-coding DNA elements in the Foxp3 gene in regulatory T-cell fate. Nature.

[10] Hsieh C.S., Lee H.M., Lio C.W. (2012). Selection of regulatory T cells in the thymus. Nat. Rev. Immunol..

[11] Smigiel K.S., Richards E., Srivastava S., Thomas K.R., Dudda J.C., Klonowski K.D., Campbell D.J. (2014). CCR7 provides localized access to IL-2 and defines homeostatically distinct regulatory T cell subsets. J. Exp. Med..

[12] Smigiel K.S., Srivastava S., Stolley J.M., Campbell D.J. (2014). Regulatory T-cell homeostasis: Steady-state maintenance and modulation during inflammation. Immunol. Rev..

[13] Tone Y., Furuuchi K., Kojima Y., Tykocinski M.L., Greene M.I., Tone M. (2008). Smad3 and NFAT cooperate to induce Foxp3 expression through its enhancer. Nat. Immunol..

[14] Feng X.H., Derynck R. (2005). Specificity and versatility in tgf-beta signaling through Smads. Annu. Rev. Cell Dev. Biol..

[15] Chi H. (2012). Regulation and function of mTOR signalling in T cell fate decisions. Nat. Rev. Immunol..

[16] Powell J.D., Delgoffe G.M. (2010). The mammalian target of rapamycin: Linking T cell differentiation, function, and metabolism. Immunity.

[17] Delgoffe G.M., Kole T.P., Zheng Y., Zarek P.E., Matthews K.L., Xiao B., Worley P.F., Kozma S.C., Powell J.D. (2009). The mTOR kinase differentially regulates effector and regulatory T cell lineage commitment. Immunity.

[18] Ma X.M., Blenis J. (2009). Molecular mechanisms of mTOR-mediated translational control. Nat. Rev. Mol. Cell. Biol..

[19] Shi H., Chapman N.M., Wen J., Guy C., Long L.Y., Dhungana Y., Rankin S., Pelletier S., Vogel P., Wang H. (2019). Amino Acids License Kinase mTORC1 Activity and Treg Cell Function via Small G Proteins Rag and Rheb. Immunity.

[20] Zeng H., Yang K., Cloer C., Neale G., Vogel P., Chi H. (2013). mTORC1 couples immune signals and metabolic programming to establish T(reg)-cell function. Nature.

[21] Huang H.L., Long L.Y., Zhou P.P., Chapman N.M., Chi H.B. (2020). mTOR signaling at the crossroads of environmental signals and T-cell fate decisions. Immunol. Rev..

[22] Han J.M., Patterson S.J., Speck M., Ehses J.A., Levings M.K. (2014). Insulin inhibits IL-10-mediated regulatory T cell function: Implications for obesity. J. Immunol..

[23] Wu D., Wong C.K., Han J.M., Orban P.C., Huang Q., Gillies J., Mojibian M., Gibson W.T., Levings M.K. (2020). T reg-specific insulin receptor deletion prevents diet-induced and age-associated metabolic syndrome. J. Exp. Med..

[24] Li Y., Lu Y., Lin S.H., Li N., Han Y., Huang Q., Zhao Y., Xie F., Guo Y., Deng B. (2021). Insulin signaling establishes a developmental trajectory of adipose regulatory T cells. Nat. Immunol..

[25] DiToro D., Harbour S.N., Bando J.K., Benavides G., Witte S., Laufer V.A., Moseley C., Singer J.R., Frey B., Turner H. (2020). Insulin-Like Growth Factors Are Key Regulators of T Helper 17 Regulatory T Cell Balance in Autoimmunity. Immunity.

[26] Myers M.G., Sun X.J., White M.F. (1994). The IRS-1 signaling system. Trends Biochem. Sci..

[27] Copps K.D., White M.F. (2012). Regulation of insulin sensitivity by serine/threonine phosphorylation of insulin receptor substrate proteins IRS1 and IRS2. Diabetologia.

[28] Hakuno F., Takahashi S.I. (2018). IGF1 receptor signaling pathways. J. Mol. Endocrinol..

[29] Machado-Neto J.A., Fenerich B.A., Rodrigues Alves A.P.N., Fernandes J.C., Scopim-Ribeiro R., Coelho-Silva J.L., Traina F. (2018). Insulin Substrate Receptor (IRS) proteins in normal and malignant hematopoiesis. Clinics.

[30] Mardilovich K., Pankratz S.L., Shaw L.M. (2009). Expression and function of the insulin receptor substrate proteins in cancer. Cell Commun. Signal..

[31] Gual P., Le Marchand-Brustel Y., Tanti J.F. (2005). Positive and negative regulation of insulin signaling through IRS-1 phosphorylation. Biochimie.

[32] Li D.Y., Xiong X.Z. (2020). ICOS(+) Tregs: A Functional Subset of Tregs in Immune Diseases. Front. Immunol..

[33] Tian J., Zhang B., Rui K., Wang S. (2020). The Role of GITR/GITRL Interaction in Autoimmune Diseases. Front. Immunol..

[34] Malek T.R., Castro I. (2010). Interleukin-2 receptor signaling: At the interface between tolerance and immunity. Immunity.

[35] Qureshi O.S., Zheng Y., Nakamura K., Attridge K., Manzotti C., Schmidt E.M., Baker J., Jeffery L.E., Kaur S., Briggs Z. (2011). Trans-Endocytosis of CD80 and CD86: A Molecular Basis for the Cell-Extrinsic Function of CTLA-4. Science.

[36] Walker L.S.K. (2013). Treg and CTLA-4: Two intertwining pathways to immune tolerance. J. Autoimmun..

[37] Passerini L., Allan S.E., Battaglia M., Di Nunzio S., Alstad A.N., Levings M.K., Roncarolo M.G., Bacchetta R. (2008). STAT5-signaling cytokines regulate the expression of FOXP3 in CD4+CD25+ regulatory T cells and CD4+CD25- effector T cells. Int. Immunol..

[38] Davey H.W., Xie T., McLachlan M.J., Wilkins R.J., Waxman D.J., Grattan D.R. (2001). STAT5b is required for GH-induced liver IGF-I gene expression. Endocrinology.

[39] Woelfle J., Billiard J., Rotwein P. (2003). Acute control of insulin-like growth factor-I gene transcription by growth hormone through Stat5b. J. Biol. Chem..

[40] Liu H., Yao S., Dann S.M., Qin H., Elson C.O., Cong Y. (2013). ERK differentially regulates Th17- and Treg-cell development and contributes to the pathogenesis of colitis. Eur. J. Immunol..

[41] Krook A., Zierath J.R. (2009). Specificity of insulin signalling in human skeletal muscle as revealed by small interfering RNA. Diabetologia.

[42] Michalek R.D., Gerriets V.A., Jacobs S.R., Macintyre A.N., MacIver N.J., Mason E.F., Sullivan S.A., Nichols A.G., Rathmell J.C. (2011). Cutting edge: Distinct glycolytic and lipid oxidative metabolic programs are essential for effector and regulatory CD4+ T cell subsets. J. Immunol..

[43] Multhoff G., Vaupel P. (2021). Lactate-avid regulatory T cells: Metabolic plasticity controls immunosuppression in tumour microenvironment. Signal Transduct. Target Ther..

[44] Gocher A.M., Handu S., Workman C.J., Vignali D.A.A. (2020). Interferon gamma production by regulatory T cells is required for response to cancer immunotherapy. J. Immunol..

[45] Overacre-Delgoffe A.E., Chikina M., Dadey R.E., Yano H., Brunazzi E.A., Shayan G., Horne W., Moskovitz J.M., Kolls J.K., Sander C. (2017). Interferon-gamma Drives Treg Fragility to Promote Anti-tumor Immunity. Cell.

[46] Zhang X., Luo Y., Ding X.F., Wang C.Q., Yang X.X., Liu M.L. (2018). Adipocyte mTORC1 Suppresses Treg Cell Development and Browning of White Adipose Tissue through CRTC2/COX-2/PGs Pathway. Diabetes.

[47] Burhans M.S., Hagman D.K., Kuzma J.N., Schmidt K.A., Kratz M. (2019). Contribution of Adipose Tissue Inflammation to the Development of Type 2 Diabetes Mellitus. Compr. Physiol..

[48] Lee W.H., Jang S.W., Kim H.S., Kim S.H., Heo J.I., Kim G.E., Lee G.R. (2019). BATF3 is sufficient for the induction of Il9 expression and can compensate for BATF during Th9 cell differentiation. Exp. Mol. Med..

