# Supplementary material for: Insulin Receptor Substrate 1 Signaling Inhibits Foxp3 Expression and Suppressive Functions in Treg Cells through the mTORC1 Pathway
Source: Int J Mol Sci. 2023 Jan 29;24(3):2551. doi: 10.3390/ijms24032551 (PMC9917118; doi:10.3390/ijms24032551)
Supplement: Supplementary file 1 [file ijms-24-02551-s001.zip › ijms-2126139-supplementary.pdf]

Supplementary Table S1. Primers used in the study.

Primer name	Sequences (5´→3´)
Foxp3 Forward primer	CCCAGGAAAGACAGCAACCTT
Foxp3 Reverse primer	TTCTCACAACCAGGCCACTTG
Foxp3 probe	ATCCTACCCACTGCTGGCAAATGGAGTC
Gapdh Forward primer	CAATGTGTCCGTCGTGGATCT
Gapdh Reverse primer	GTCCTCAGTGTAGCCCAAGATG
Gapdh probe	CGTGCCGCCTGGAGAAACCTGCC
Irs1 Forward primer	CGATGGCTTCTCAGACGTG
Irs1 Reverse primer	CAGCCCGCTTGTTGATGTTG
Ifng Forward primer	GGATGCATTCATGAGTATTGC
Ifng Revers primer	CCCTTTTCCGCTTCCTGAGG
Il2ra(Cd25) Forward primer	AACCATAGTACCCAGTTGTCGG
Il2ra(Cd25) Reverse primer	TCCTAAGCAACGCATATAGACCA
Tnfrsf18 (Gitr) Forward primer	CGCGGGGAGCAGACAGAAGAA
Tnfrsf18 (Gitr) Reverse primer	GGCCCAAAGACCCTACTCCAACAG
Cd278 (Icos) Forward primer	TGACCCACCTCCTTTCAAG
Cd278 (Icos) Reverse primer	TTAGGGTCATGCACACTGGA
Cd152(CTLA4) Forward primer	GCTTCCTAGATTACCCCTTCTGC
Cd152(CTLA4) Forward primer	CGGGCATGGTTCTGGATCA
